# Community-based monitoring: people's health in people's hands

**Published:** 2022-09-20

**Authors:** Kriti Shukla

**Affiliations:** Research Associate: Centre for Health Outcomes Research and Economics, Indian Institute of Public Health, Hyderabad, India.

**Community-based monitoring of health services can put people back in control of their health and the health services they need.**

**Figure:**
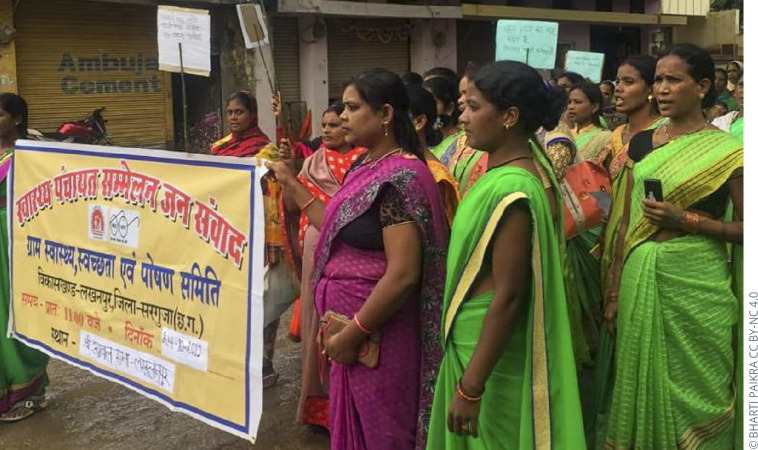
ASHA workers rally to inform village residents and committee members about the time and location of their village health, sanitation and nutrition committee meeting (see panel on page 15). india

In Thane, India, 17-year-old Ankita, who has drug-resistant tuberculosis (DR TB), required an essential tuberculosis drug, administered orally. However, due to a lack of stock at the Directly Observed Treatment Short-course (DOTS) centre, where the drug is provided free of cost to all tuberculosis (TB) patients, she was put on alternative drugs that had to be injected. These are very painful and have serious side effects, including the risk of hearing loss. Not having enough money to pay for a private prescription of her usual medication, and not knowing what to do, Ankita contacted Ganesh – a TB supporter and activist – for help. In India, TB activists and supporters have formed a countrywide network to help TB patients and to advocate for issues around TB. They stay in constant contact with each other, and with patients, by phone and WhatsApp. Community TB champions visit DOTS centres and connect with patients, making it easy for patients, such as Ankita to reach out to peer supporters like Ganesh.

Next, Ganesh reached out to other TB activists and supporters. With many of them having had similar experiences of drug shortages, and realising that this was a wider issue, the TB support groups initiated a community-based monitoring system. They collected information on drugs that were out of stock, diagnostic kits that were unavailable, and the number of TB patients requiring these services. They shared this information with the government and asked for action to be taken; they also contacted the media to highlight the challenges of TB treatment. As a result of their constant monitoring and advocacy, the government promised to deal with drug shortages. In 2022, officers from the government's TB programme also began to convene monthly meetings with activists, patients, TB survivors, and non-governmental organisations (NGOs) to discuss the needs of TB patients and the challenges of treatment.

The actions taken by Ganesh and the TB activists to support Ankita (and others in a similar situation), are an example of **community-based monitoring** – a way for patients and the wider community to check whether health programmes and health services are effective, are accessible to everyone, are of good quality and have the desired impact.

The key principle of community-based monitoring is that the **community** decides what to monitor and how to act upon the data collected.

In the example above, the TB activist community:

- continually monitored and documented the availability of essential TB medicines and diagnostic tests
- kept in touch with service users (TB patients) to understand their concerns
- shared this information with decision makers (e.g., government) so they could act quickly to prevent future medicine shortages and to provide access to treatment for all.

Community-based monitoring can provide an efficient feedback mechanism and ensures that service providers are accountable to the service users (the public or community). This shifts the focus away from the providers and their work, to the community and their needs, and how well these are being met. In other words, community-based monitoring helps to create a people-oriented public health delivery system. It works both ways: community-based monitoring also helps communities to understand the challenges faced by the government and/or service providers. This can result in community members and decision makers working collaboratively to solve issues as they arise in real time. All of this helps to foster communities' active participation in health care and empowers them to claim their rights to health.

## How does community-based monitoring relate to eye care?

Patient- or community-led advocacy and monitoring may not yet be well established in all areas of eye care. However, some of the vital components of eye care – such as vitamin A distribution, nutrition, environmental safety, and essential medicine availability – are already being monitored by communities in various countries, as are some of the key determinants of eye health such as age, gender, poverty, and disability.

In addition, monitoring is especially important in conditions such as glaucoma, diabetic eye disease, trachoma, and pediatric eye care.

Eye care organisations, on their own or in partnership with grassroots organisations and civil society organisations (CSOs), can support or offer training to communities to help them actively and independently monitor and collect data regarding eye care services. Some examples are:

- providing community score cards at eye health facilities (see below) and carrying out patient satisfaction surveys
- establishing strong and streamlined grievance mechanisms
- facilitating social audits of eye care services.

All of these approaches can lead to better provision of services and ensure that communities have more say in the way services are provided.

**Figure:**
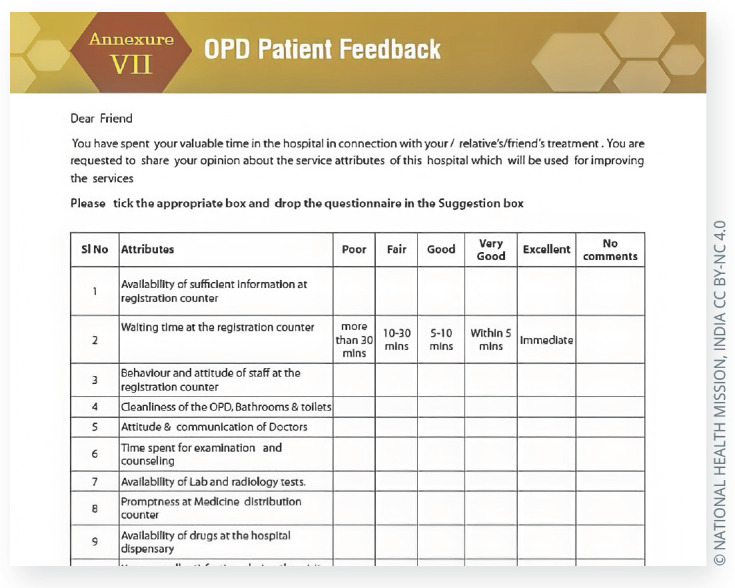
Score cards and surveys can be displayed in hospitals and clinics where patients can find them easily. Community organisations can encourage patients to fill in these forms, collect them, and note any issues that need to be resolved by the health provider. india

## How community-based monitoring works

**Figure 1:**
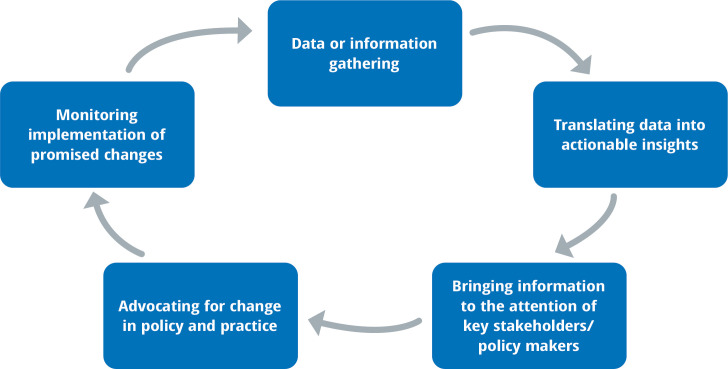
Community-based monitoring can be viewed as a five-stage process

Community-based monitoring can be viewed as a five-stage process (Figure 1),^1^ with communities taking the decisions and leading the work at each stage, supported by community service organisations, health activists or movements, and by non-profit health organisations.

1. **Data or information gathering.** This can involve directly observing conditions and services; interviewing service users; conducting focus group discussions, public hearings, and meetings of local governing bodies; carrying out participatory appraisals and social audits; and so on. Organisations can help communities learn about and adopt suitable data collection and case documentation methods.
2. **Analysing information and translating it into actionable insights.** Communities can identify what is working well and the gaps that need to be addressed, such as identifying which primary health care facilities have long queues and over-full waiting rooms; where there are medicine shortages and/or inadequate equipment; where appointments are often cancelled; and so on. Organisations can also help communities to understand their own health status, their entitlement to health, and their rights relating to health.
3. **Disseminating findings.** This involves sharing the insights gained with a wide range of groups and individuals (including health providers, government, officials and policy makers) from the local to national level. Health providers from clinic level through to the ministry of health can support communities by being willing to listen to them and providing opportunities for the community to give feedback.
4. **Advocating for change.** It is essential to start advocacy at the facility level by making facility managers aware of issues, persuading them to address the issues, and holding them accountable. Health providers can support this process by responding positively to any issues raised and working with the community to develop solutions. To communicate advocacy messages to policy makers and hold them to account, the media – both print and electronic – can be a powerful resource, as in the TB example. The media can also play an important role in educating the public and can be engaged to raise the visibility of the problems faced by communities – which can also prompt policy makers to act.
5. **Monitoring the implementation of promised changes.** Communities can monitor the status of the commitments made by health providers and policy makers. For instance, a local commitment to build a new vision centre or eye clinic may not be followed through, perhaps due to a shortage of staff. This would require further advocacy and action.

The reason for engaging communities in health care is rooted in the understanding that health is also influenced by various non-medical determinants: the conditions in which people are born, grow, work, and live; their age, gender, behaviour, and lifestyle; and political conditions. It's very difficult for health providers to know whether the available services are meeting everyone's needs – it is therefore vital to involve communities in monitoring how well their needs are being met. For example, women or people with disabilities will know best what challenges they face when they access services, so they are the best people to monitor how well access is going for them and to give that feedback to eye care providers via community groups or forums.

**An example of community-based monitoring**

A useful example in the Indian health system is the National Health Mission's community-based monitoring framework.^2^ This interactive system of monitoring is designed to keep communities in the centre, with the aim of increasing public participation in, and monitoring of, health care – from the village level to the level of the state.

**Figure 2:**
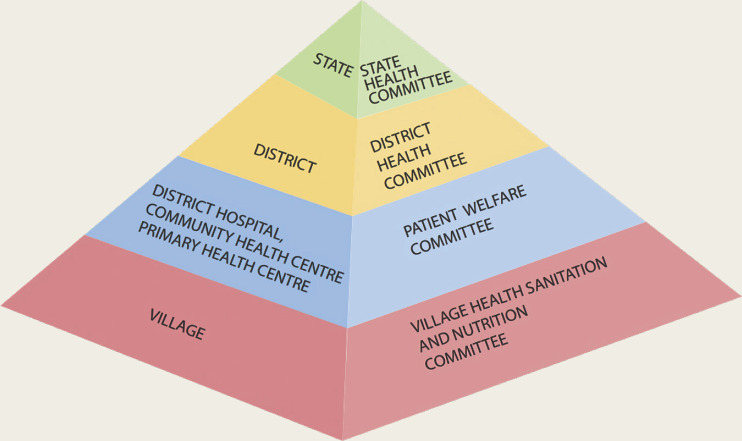
Community-based monitoring committees at different levels in the health system of India

There is a monitoring committee for each level of the system (see Figure 2), Each committee includes representatives from the community such as local self-elected members (panchayat members), community health workers (ASHAs), Anganwadi workers/ Integrated Child Development Services workers, community-based women's collectives, members of patient welfare committees, NGO representatives, and medical officers.

The monitoring committee at each level reviews the functioning of the health care facilities at that level and work to streamline grievance procedures, collect reports, and take action to improve patient care and welfare. Committees report issues that require further intervention to the next level up; for example, from a district health committee to the state health committee.

Rather than relying solely on reports, each of the committees interact directly with the community in their area by conducting public hearings every six months. At these hearings, they receive feedback from the community regarding the quality and accessibility of services, and about patients' experience of these services.

A recent concern about the unavailability of diabetes medication demonstrates how the system works. Several village members attending a primary health centre (PHC) were told that their diabetes medication (usually provided free of cost) would be unavailable for several days due to issues with the supply chain. As a result, they had to purchase their own medicine from a private pharmacy, and some stopped using the medicine as they couldn't afford it. After discussing the issue at a village meeting, a representative from the village attended a meeting of PHC's patient welfare committee to explain what had happened.

The patient welfare committee dealt with the immediate challenge by using their own funds to supply the medicine to patients in the short term, so they would not have to face further inconvenience or cost. The committee also reported the procurement issue to the district health committee so they could take action to avoid this same issue happening again.

Community-based monitoring is a participatory process which requires resources and support from other grassroots organisations and social activists. It also requires evidence gathering and advocacy. Therefore, concerted efforts are required to build capacity and empower communities to engage with public health systems so they can realise their right to health.

When health providers and officials at various levels support community-based monitoring, they are effectively putting people's health in people's hands – where it belongs.

## References

[1] Community-led monitoring of health services: building accountability for HIV service quality [Internet]. Available from: **https://bit.ly/3wqFxKD**

[2] National Health Mission [Internet]. https://bit.ly/3Rcx06j.

